# Bibliometric analysis of depression in post-stroke patients

**DOI:** 10.1186/s12245-024-00725-y

**Published:** 2024-10-04

**Authors:** Dehao Zheng, Sydney Vaughn, Murdoc Gould, Latha Ganti

**Affiliations:** 1 Trinity Preparatory School, Winter Park, Fl USA; 2 Brown University, Providence, Rhode Island, USA; 3 Birkbeck, University College London, London, UK; 4 Orlando College of Osteopathic Medicine, Winter, FL 34787 USA; 5 Warren Alpert Medical School of Brown University, Providence, RI 02903 USA

**Keywords:** Post-stroke depression, Bibliometric analysis

## Abstract

**Introduction:**

Stroke is a life-threatening condition that increasingly damages cerebral tissue over time and can lead to serious post-effects, including depression, which can hinder a patient’s recovery from stroke and reduce quality of life. This paper aims to analyze the global research landscape of post-stroke depression (PSD) between the years 1900 to 2024 using bibliometric analysis.

**Methods:**

The data used in this analysis was collected from the Web of Science Core Collection (WoSCC). An advanced search was performed using the keywords, “stroke” and “depression,” on July 8th, 2024. From the Web of Science, bibliometric data was then extracted and analyzed in VOSviewer through four categories: countries, number of publications, keywords, and journals.

**Results:**

The bibliometric analysis resulted in 2,289 publications from the year 1900 to 2024. A gradual increase in the number of publications on post-stroke depression over the study period was observed. China was found to be the leading country for publications and funding on PSD, with the United States following in second. The top keywords included: “stroke,” “depression,” and “poststroke depression.” The Journal of Stroke had the highest number of publications on depression in post-stroke patients.

**Conclusion:**

This study provides an overview of the current trends in articles published on PSD. Depression is an important topic to be considered in post-stroke patients due to its negative effects on post-stroke recovery and reduced quality of life, necessitating a call to attention and support for future research in this field. With continued research efforts led by the United States and China, improved treatments for patients with post-stroke depression can be implemented.

## Introduction

Stroke is the second leading cause of death, the third leading cause of disability, and the number one leading cause of hospitalization for neurologic disease [1]. There are two classifications of stroke: hemorrhagic and ischemic. Common stroke risk factors include: hypertension, diabetes, and cigarette smoking. Risk factors for ischemic stroke include: coronary heart disease, atrial fibrillation, heart valve disease, carotid artery disease, hypothyroidism, obstructive sleep apnea [2] Risk factors for hemorrhagic stroke include: hypertension, male sex, Asian ethnicity, alcohol intake, low levels of total serum cholesterol, genetics, use of anticoagulants, and use of sympathomimetic drugs, older age, and cerebral amyloid angiopathy (which is directly related to age) [3].

A stroke occurs when there is a blockage of blood supply to the brain or bleeding within the brain. In both cases, cerebral tissue becomes increasingly damaged over time and can lead to serious post-effects. For example, some conditions that emerge after stroke include Alien Hand Syndrome, Aphasia (Broca’s and Wernicke’s), Anton’s Syndrome, Balint Syndrome, and more. These syndromes, respectively, affect movement, speech, sight, and focus, making it difficult for patients to carry out day-to-day tasks that they once could perform prior to the stroke. According to the Framingham study, of the 6.5 million individuals who survived a stroke and are alive today, almost 50% have moderate to severe neurological deficits, 30% are unable to walk without external help, and over 25% need assistance in their daily activities [4].

Depression is a serious mood disorder that can negatively impact day-to-day life. Symptoms of depression include feelings of sadness, hopelessness, distress, or guilt, loss of interest in previous hobbies or activities (anhedonia), decreased energy, concentration or sleeping difficulty, and changes in appetite or weight. Additionally, depression can cause one to have increased thoughts of suicide or self-harm, irritability, use of alcohol or drugs, or isolation from family and friends and responsibilities. Risk factors for depression include having a previous experience with depression, family history of depression, major negative life changes, trauma, or stress. Studies have shown an increased correlation between stroke and depression, with a stroke being deemed the major negative life change leading to cases of depression [5].

Depression in post-stroke patients is important to consider because of the various ways depression can significantly impact a person’s lifestyle. Depression can also hinder the patient’s recovery process from stroke and reduce their quality of life. According to several studies on patients who survive a stroke incident, 10–40% of stroke patients develop mild depression, while 10–25% of patients develop major depression [6]. One reason for post-stroke depression (PSD) is due to damage to certain cerebral areas that were affected by the stroke. For example, acute stroke patients with left frontal or left basal ganglia lesions had higher frequencies of depression than patients with other lesion locations [7, 8]. Another possible reason for PSD is medication. Hypertension heightens the risk for ischemic stroke. As a result, post-stroke patients with high blood pressure may be prescribed beta blockers to lower blood pressure, but may consequently have an increased risk of depression because of the medication [9]. From a non-technical standpoint, depression may also develop as a result of a major negative life change caused by post-stroke effects. Strokes can potentially result in various disabling conditions hindering some patients from performing basic activities that were once simple or in extreme cases requiring a caregiver for monitoring and assistance. As a result of sudden negative changes in lifestyle due to stroke, patients are at risk of developing depression.

A bibliometric analysis is the quantitative analysis of books, articles, or other academic publications. It is used to study the trends of the publications of a particular topic from various angles, including keywords, country of origin, journal of publication, and more. This paper presents a bibliometric analysis on depression in post-stroke patients. While the treatment and prevention of death in stroke is important, consideration for post-stroke effects is just as crucial. Through a bibliometric analysis, an overview of the current research landscape of post-stroke depression is observed.

## Methods

The data used in this analysis was collected from the Web of Science Core Collection (WoSCC). The WoSCC was selected due to its vast range of data, covering more than 34,000 journals today. Established in 1964, WoSCC is the oldest database for scientific journal publications and citations. In comparison to other databases, the WoSCC continues to be the most widely used today, enhancing its credibility. This specific database allows for various ways of analyzing data through its advanced filter functions, as well as allowing for the extraction of bibliometric information [10]. An advanced search for the keywords “stroke” and “depression” of which were both present in the article title was performed on July 8th, 2024. The search strategy, TI=(stroke and depression), was applied in order to retrieve more accurate outcomes. The search yielded 2,289 results from the year 1900 to 2024.

Bibliometric data was extracted from the Web of Science and analyzed in VOSviewer, a software tool designed for analyzing bibliometric data and producing visual and analytic maps. Using VOSviewer, all 2,289 articles were examined through three major categories: country, journal, and major keywords. Additional data on the date of article publications and funding organizations were obtained through the WoSCC itself.

## Results

**Fig. 1:**
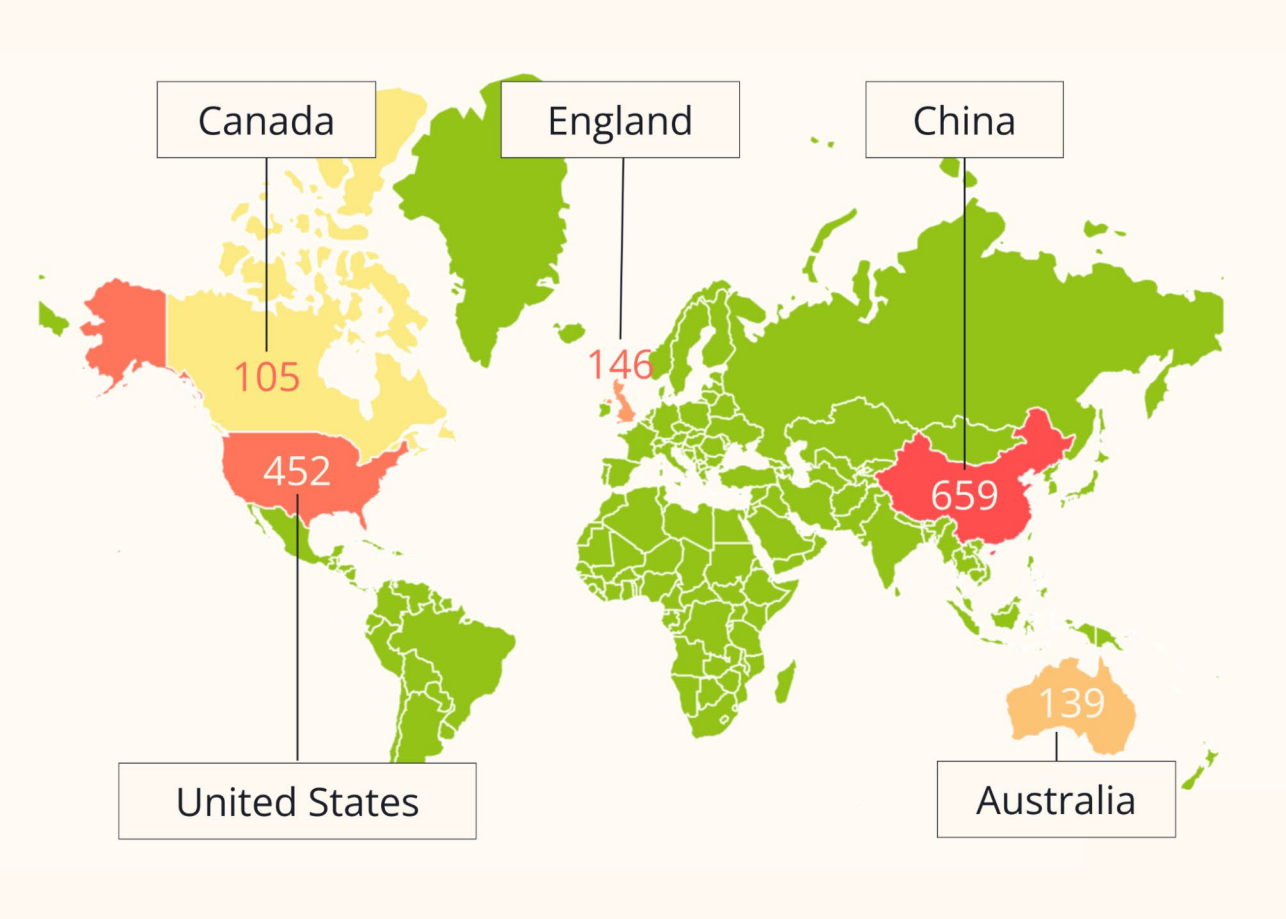
Visual of the top five countries with the most amount of publications on post-stroke depression

**Fig. 2:**
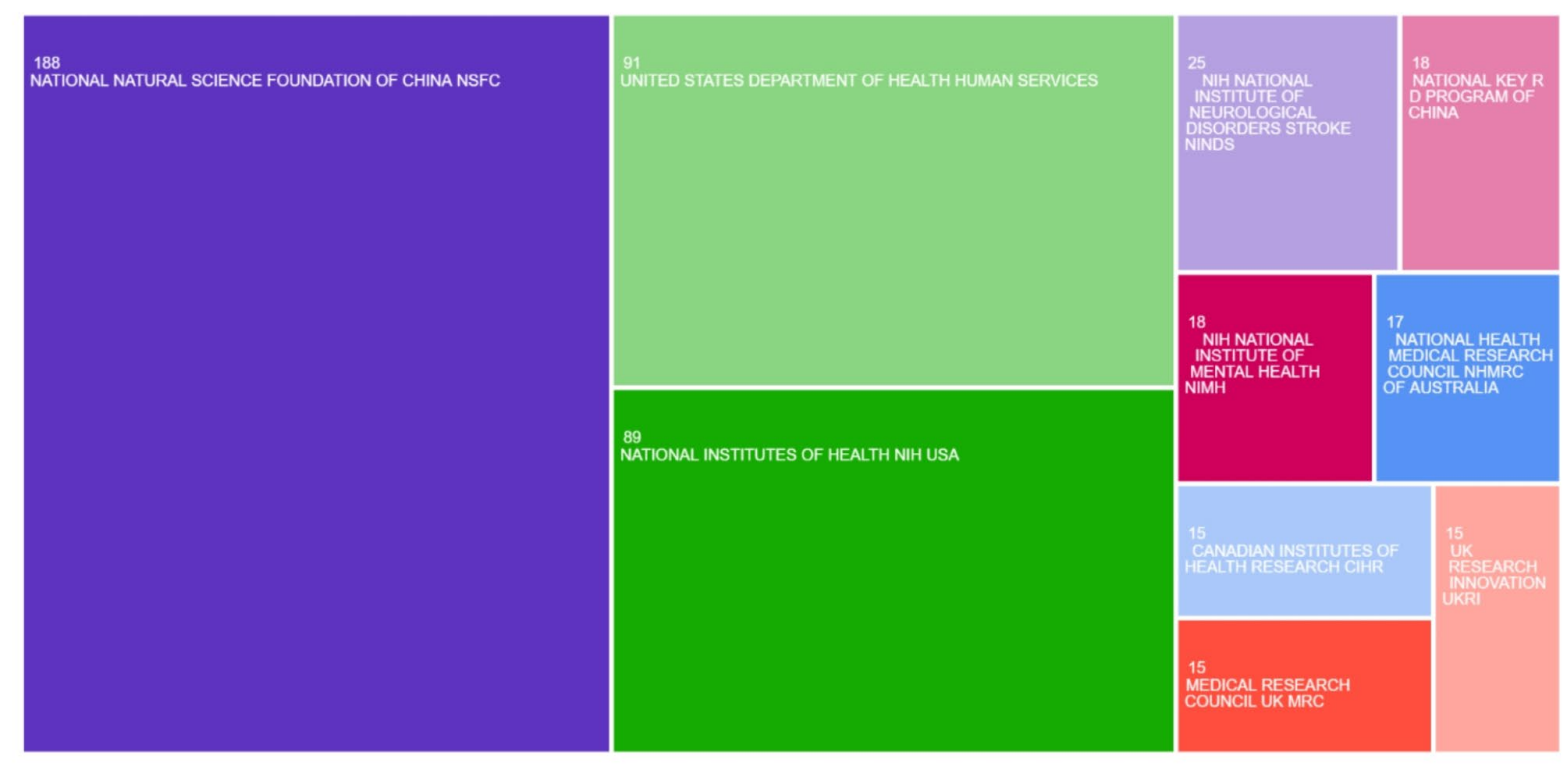
Visual of the top five funding organizations for publications on PSD

The top five most productive countries are listed below in Table 1; Fig. 1. China leads the ranking of countries for the number of publications on depression in post-stroke patients (659 publications), accounting for more than a quarter of all papers. In addition, the National Natural Science Foundation of China (NSFC) individually provided funding for the most number of publications on post-stroke depression. Following China, the United States is second for both the number of publications and funding agencies. Three organizations in the United States, which are all part of the United States government include the United States Department of Health and Human Services, the National Institutes of Health (NIH), and the National Institute of Neurological Disorders and Stroke. These three organizations collectively funded a combined 205 papers on the topic of post-stroke depression [Fig. 2].

**Table 1:** Table of the top five countries with the most amount of publications on post-stroke depression

Ranking	Country	No. of Publications	Percentage
1	People’s Republic of China	659	28.777%
2	USA	452	20.087%
3	England	146	6.638%
4	Australia	139	6.114%
5	Canada	105	4.672%

**Fig. 3:**
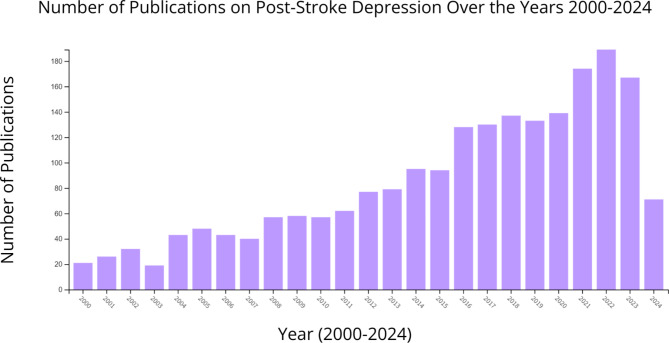
Graph showcasing the number of publications on PSD over the years 2000–2024

The number of articles on post-stroke depression has been steadily increasing over time. Figure 3 displays the number of publications per year within the timeframe 2000–2024 [Fig. 3]. The highest number of publications was in 2022 with 189 documents, accounting for 8.253% of all publications.

**Fig. 4:**
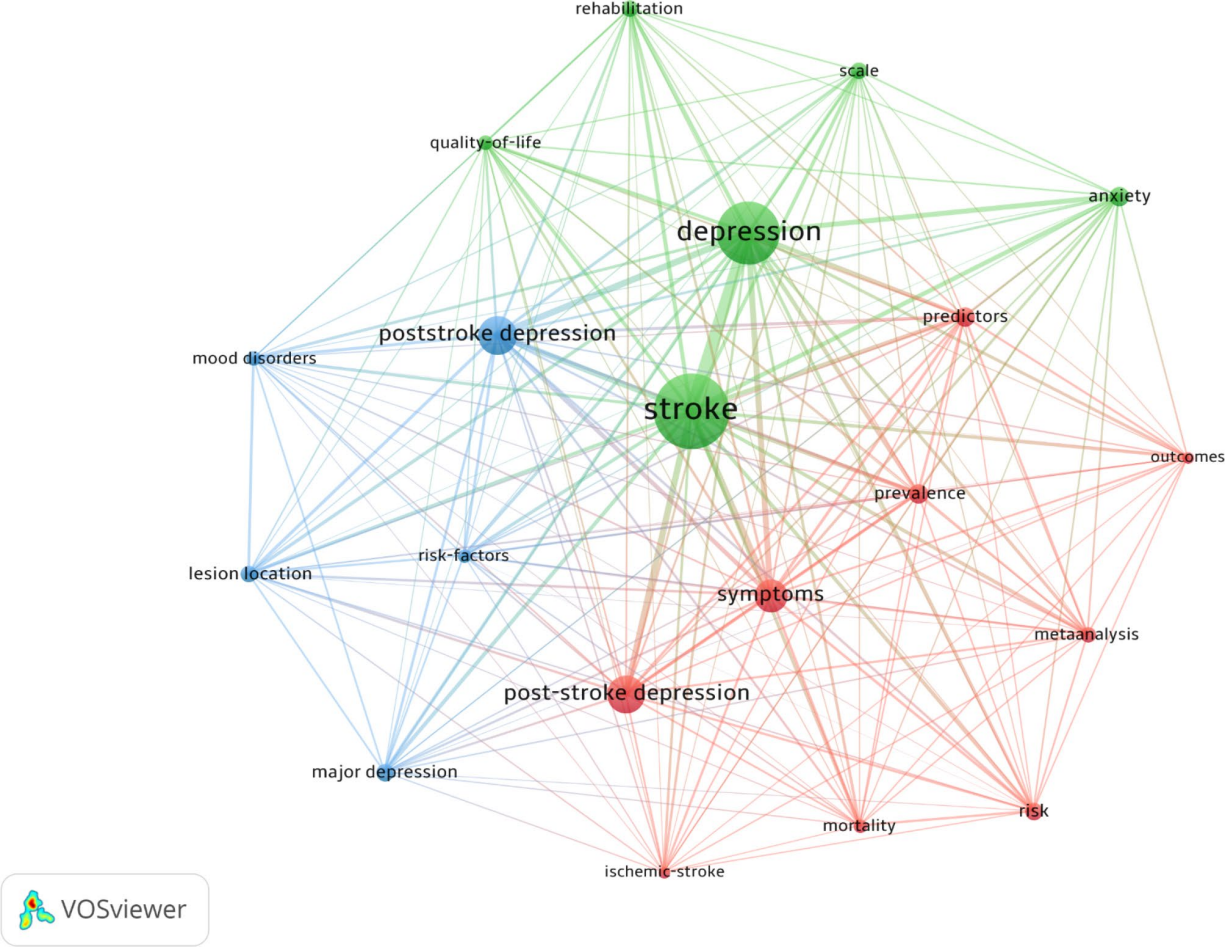
Visual of the top 20 keywords used in post-stroke depression publications

Table 2; Fig. 4 lists the top 20 keywords used in PSD papers. Frequently utilized keywords can highlight the common or most popular topics of the various publications on post-stroke depression.

**Table 2:** Table of the top 20 keywords used in post-stroke depression publications

Ranking	Keyword	Occurrences	Ranking	Keyword	Occurrences
1	Stroke	869	11	Lesion Location	167
2	Depression	707	12	Scale	163
3	Poststroke Depression	414	13	Rehabilitation	159
4	Post-stroke Depression	406	14	Meta-Analysis	153
5	Symptoms	343	15	Quality-of-life	139
6	Anxiety	198	16	Mood Disorders	139
7	Prevalence	192	17	Mortality	135
8	Predictors	191	18	Risk-factors	132
9	Risk	172	19	Outcomes	116
10	Major Depression	170	20	Ischemic-stroke	114

The 2,289 articles were published in 603 different journals. The most popular journals with the highest amount of documents on post-stroke depression published are displayed in Table 3.

**Table 3:** Top five ranking journals for publications on PSD

Ranking	Journal	No. of Publications	Percentage
1	Stroke	187	8.170%
2	International Journal of Stroke	102	4.456%
3	Journal of Affective Disorders	59	2.578%
4	Cerebrovascular Diseases	48	2.097%
5	European Journal of Neurology	48	2.097%

## Discussion

### Countries

China and the United States are the top two most productive countries in publications on post-stroke depression. Their considerable interest in this topic is most likely due to their high prevalence in both stroke and depression. In China, stroke is the leading cause of death, affecting 2.6% of the population in 2020 with 17.8 million prevalent cases [10]. Stroke is the fifth leading cause of death in the United States, affecting around 795,000 people each year [11]. Additionally, there are an estimated 54 million people in China who suffer from depression, which is approximately 4.2% of the population, according to the World Health Organization (WHO) [12]. Although the prevalence of depression (4.2%) is in the average range, China has one of the most people affected by depression due to its large population. In the USA, there are about 17 million cases of depression, making up 5.9% of the population [12]. Although there are fewer people with depression in the U.S. than in China, the United States has a lower population, so the prevalence of depression is higher. In both cases, stroke and depression have high prevalences and are very important subjects in both countries.

Another reason why China and the United States have the most studies on post-stroke depression is due to funding. Out of the top five funding agencies for publications on this topic, two were from China (The National Natural Science Foundation of China (NSFC) and National Key Technologies R&D Program), and three were from the USA (the United States Department of Health and Human Services, National Institutes of Health (NIH), and National Institute of Neurological Disorders and Stroke). Both China and the United States are large and wealthy countries, which allows for increased allocation of funding towards research.

### Number of articles

The number of articles on post-stroke depression has been steadily growing over the years, which is most likely due to the increasing cases of stroke, and therefore increasing incidences of depression. According to an analysis done using the Global Burden of Disease (GBD) 2019 study, the change in the prevalence of stroke in the general population increased by about 60% since 1900. Incidence, mortality, and disability-adjusted life years also increased by about 20% [13]. There are various reasons behind the increasing prevalence of stroke, including an expanding human population, longer life expectancy, and rising incidence of diabetes and high blood pressure. Because the human population is increasing, there will naturally be more stroke cases than in previous years. Life expectancy has also increased due to improvements in healthcare and accessibility. The older a person is, the more susceptible they are to stroke, especially individuals above 75 years of age [2]. Lastly, the incidence of diabetes and hypertension is rising due to increased accessibility of unhealthy foods and poor lifestyle habits, and as major risk factors for stroke, the increasing prevalence may also correspond to more stroke cases [14–16].

The increase in articles also means that post-stroke depression is gaining attention, which is beneficial because the elderly population has the highest rates of stroke, but also faces greater risks and effects of depression. Depression is often undiagnosed in the elderly because the vague physical symptoms of depression mimic many other coexisting medical conditions that come with old age [17]. One systematic review/meta-analysis of depression among the elderly found that disability was 1 of 5 key risk factors that were identified within the senior population [18]. Older people with depression have also been shown to have increased suicide rates. Of those older than 74 years who died from suicide, 80% had depressive syndromes [19].Therefore depression is a serious topic and needs to be considered in all people who face an increased risk of this condition.

### Keywords

Analyzing the top keywords creates an overview of the most popular topics being written about post-stroke depression. Besides the most obvious keywords, other terms provide a deeper insight into the type of papers being published. For example, some of the top 20 keywords such as “symptoms,” “anxiety,” and “predictors” demonstrate a high prevalence of papers on the detection of depression in post-stroke patients. The detection of depression is crucial to efficient diagnosis and the treatment of patients, including prescribing antidepressants or therapy. Depression can hinder the patient’s recovery process from stroke and quality of life, so it is important to treat it as soon as possible. Some other keywords such as “risk,” “risk factors,” “ischemic stroke,” and “lesion location” suggest a large amount of studies being done on potential risk factors for depression, including factors in the brain itself. Results have varied between studies, so there are no solid differences in depression rates between ischemic and hemorrhagic strokes [20–22]. However, some studies have found data supporting the relevance of lesion location, in that patients with left-sided cerebral lesions, specifically the left frontal or left basal ganglia, have a slightly greater risk of depression [6, 7]. Discovering the cause of the risk for depression can help with early prevention or detection. This area of study is definitely an important field for future research.

### Journals

There were 603 journals in total, holding 2,289 articles on post-stroke depression. While China and the United States had the most number of publications, the journals they were published in were not limited to a specific journal belonging to their respective country. Instead, the Journal of Stroke and the International Journal of Stroke were the two most notable journals, with 187 and 102 publications respectively. The prominence is likely because they are international journals and offer open access publications. In addition, these two journals do not require any fees to submit or publish articles. However, the popularity of the Journal of Stroke may also be related to its increased interest in studies relating to stroke in the Asian population [22]. China, the primary contributor to PSD publications, has likely contributed many articles to the Journal of Stroke due to its high interest in stroke research concerning the Chinese population.

### Limitations

Because data collected for analysis was confined to the Web of Science, other academic databases were excluded which may have led to an incomplete collection of data and holes in the bibliometric analysis. In addition, using the advanced search function for “stroke” and “depression” both appearing in the publication title may potentially miss some articles that only mention one term or neither term in the title but still discuss PSD within the paper itself. However, this advanced search was necessary to rule out articles that pertained to only stroke or to only depression. Finally, because this analysis was done in July 2024, data for the number of publications in 2024 was incomplete, and therefore an increase or decrease in 2024 publication rates in comparison to other years could not be observed.

## Conclusion

Stroke is a crucial condition that can significantly impact the patient, which is why it is important to analyze the post effects, especially depression. Through this bibliometric analysis, it is evident that the number of publications for post-stroke depression has progressively increased; China is the leading country for publications and funding on PSD, with the United States following in second; the top 20 keywords implied a large amount of research on the diagnosis and risk factors for depression in post-stroke patients; the Journal of Stroke was the top journal with the most number of publications.

In summary, this bibliometric analysis reveals a growing interest in post-stroke depression. Depression is an important topic to be considered in post-stroke patients because of its negative effects on patients after stroke, including hindering recovery and reducing quality of life, which necessitates a call to attention and support for future research.

## Data Availability

No datasets were generated or analysed during the current study.
